# Synergistic effect of teclistamab with PD-1 inhibition: a case of acute interstitial nephritis with dual immunotherapy

**DOI:** 10.1093/ckj/sfae425

**Published:** 2024-12-20

**Authors:** Arjunmohan Mohan, Elena-Bianca Barbir, Loren Herrera Hernandez, Nelson Leung, Sandra M Herrmann

**Affiliations:** Division of Nephrology and Hypertension, Department of Medicine, Mayo Clinic, Rochester, MN, USA; Division of Nephrology and Hypertension, Department of Medicine, Mayo Clinic, Rochester, MN, USA; Division of Anatomic Pathology, Mayo Clinic, Rochester, MN, USA; Division of Nephrology and Hypertension, Department of Medicine, Mayo Clinic, Rochester, MN, USA; Division of Hematology, Mayo Clinic, Rochester, MN, USA; Division of Nephrology and Hypertension, Department of Medicine, Mayo Clinic, Rochester, MN, USA

**Keywords:** acute interstitial nephritis, bispecific antibodies, immune checkpoint inhibitors, multiple myeloma, teclistamab

## Abstract

We report a case of acute interstitial nephritis (AIN) in a 68-year-old male with squamous cell carcinoma (SCC) and relapsed/refractory multiple myeloma (RRMM) who developed acute kidney injury (AKI) shortly after starting teclistamab for RRMM. Despite stable renal function on immune checkpoint inhibitor (ICI) therapy for SCC, proton pump inhibitors and trimethoprim-sulfamethoxazole for 3 years, AKI only occurred post-teclistamab initiation. Biopsy-confirmed AIN with serum creatinine improved only after pulse-dose steroids. This case highlights a potential synergistic nephrotoxic effect of teclistamab and ICIs, warranting further investigation into teclistamab's renal safety.

## INTRODUCTION

Teclistamab, a bispecific T-cell engager (BiTE), is approved for the treatment of relapsed or refractory multiple myeloma (RRMM) [1]. Due to its recent introduction, safety data—particularly regarding its use with other immunotherapies, remain limited. We report a case of acute interstitial nephritis (AIN), requiring pulse-dose steroids for successful treatment, occurring shortly after initiating teclistamab in a patient with RRMM who had been stable on immune checkpoint inhibitor (ICI) therapy for 3 years for oropharyngeal squamous cell carcinoma (SCC).

## CASE DESCRIPTION

**Figure 1:**
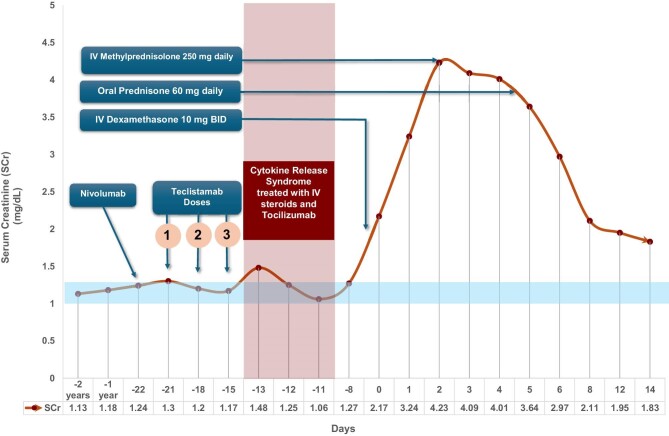
Timeline of events and SCr trend. Day 0 corresponds to the day of AKI diagnosis.

A 68-year-old male with immunoglobulin G kappa multiple myeloma (MM) and SCC presented with excessive drowsiness, altered behaviour and impaired executive function. He was on nivolumab for 3 years for SCC along with AIN-associated drugs like proton pump inhibitors (PPIs) and trimethoprim-sulfamethoxazole (TMP-SMX) and maintained stable renal function. Three weeks (day −21) prior to presentation, teclistamab was started for RRMM. Acute kidney injury (AKI) was noted on presentation (day 0), with a serum creatinine (SCr) of 2.17 mg/dl (baseline SCr 1.0–1.2 mg/dl) (Fig. 1). The initial diagnostic workup is detailed in Supplementary Table S1. Elevated urinary retinol binding protein:creatinine ratio (URBP:Cr) of 21 212 µg/g raised suspicion for AIN. Two weeks prior to this presentation he had developed grade 2 cytokine release syndrome (CRS) with fever, hypoxaemia, AKI and transaminitis. Clinical and biochemical manifestations resolved completely with tocilizumab and glucocorticoids.

At the time of nephrology consultation, he was already on empiric corticosteroids [intravenous (IV) dexamethasone 10 mg twice a day] for suspected immune effector cell-associated neurotoxicity syndrome from teclistamab. Teclistamab was held. Despite steroids, SCr worsened to 4.23 mg/dl. TMP-SMX and PPI were held due to suspected AIN and pulse-dose methylprednisolone (250 mg IV daily) was initiated. A kidney biopsy revealed AIN with severe focal tubulitis with rare kappa light chain-restricted casts and the presence of acute tubular injury (ATI) (Supplementary Fig. S1). Staining for plasmacytes (CD38 and CD138), CD20 and B-cell maturation antigen (BCMA) was negative. After a rapid improvement in SCr with pulse-dose steroids, a tapering dose of prednisone was initiated with eventual improvement in SCr to 1.8 mg/dl within 2 weeks (Fig. 1). The patient did not require renal replacement therapy. Unfortunately, he later developed a plasma cell leukaemia and was transitioned to hospice care.

## DISCUSSION

We report the first biopsy-confirmed case of AIN in a patient with stable renal function on nivolumab, PPI and TMP-SMX for several years who developed AKI shortly after initiating teclistamab for RRMM. Recent studies show AKI incidence with teclistamab ranges from 11 to 30%, with 3.6% experiencing grade 3–4 AKI [2, 3].

The exact mechanism of teclistamab-induced AKI remains unclear. Drug-induced AIN results from a T cell–mediated immune response, triggered by direct immunogenicity or by the drug acting as a hapten. Concomitant ICI therapy may amplify this immune response or reactivate drug-specific effector T cells [4]. Alternatively, antigen homology between SCC cells and renal tubules, amplified in a post-CRS milieu, may trigger cross-reactivity. However, this mechanism could have occurred at any point during ICI therapy.

Teclistamab, a BCMA × CD3 BiTE, recruits and activates T cells against MM cells, potentially triggering a local inflammation [2]. While the patient later developed plasma cell leukaemia, negative staining for plasmacytes makes direct infiltration unlikely. The timing of AKI after teclistamab initiation, despite prior stability of renal function for 3 years on ICI, PPI and TMP-SMX, underscores a potential synergistic effect between teclistamab and other immunotherapies in inducing AIN.

Biomarkers including elevated tumour necrosis factor-α, interleukin (IL)-6 and particularly soluble IL-2 receptor, alongside a significantly elevated URBP:Cr were suggestive but not diagnostic of ICI-AIN [5]. However, the biomarker profile was confounded by recent CRS treated with tocilizumab, which can sustain elevated IL-6 levels while elevated URBP is also observed in ATI. Although a biopsy revealed ATI and AIN, and lack of response to the initial lower dose of oral glucocorticoids (0.5–1 mg/kg) could suggest ATI, rapid improvement with pulse-dose steroids favours AIN with robust immune activation by dual immunotherapy requiring intensified therapy [4].

This case highlights the need for kidney biopsy in complex AKI cases involving novel agents to guide management. With limited safety data on teclistamab and BiTEs, further research is critical to elucidate the mechanisms of AKI, drug interactions and immunotherapy-related adverse events, optimizing the safety of cancer treatments.

## Supplementary Material

sfae425_Supplemental_File

## References

[1] US Food and Drug Administration . FDA approves teclistamab-cqyv for relapsed or refractory multiple myeloma. https://www.fda.gov/drugs/resources-information-approved-drugs/fda-approves-teclistamab-cqyv-relapsed-or-refractory-multiple-myeloma (accessed 12 September 2024).

[2] Moreau P, Garfall AL, van de Donk NWCJ et al. Teclistamab in relapsed or refractory multiple myeloma. N Engl J Med 2022;387:495–505. 10.1056/NEJMoa2203478 35661166 PMC10587778

[3] Charkviani M, Vaughan LE, Sandahl TB et al. Incidence of acute kidney injury in patients with relapsed and refractory multiple myeloma treated with teclistamab vs chimeric antigen receptor T-cell therapy. J Clin Oncol 2024;42(16 Suppl):7542. 10.1200/JCO.2024.42.16_suppl.7542

[4] Barbir EB, Kitchlu A, Herrmann SM. Immune checkpoint inhibitor-associated nephritis-treatment standard. Nephrol Dial Transplant 2024;39:1785–98. 10.1093/ndt/gfae184 39138117

[5] Farooqui N, Zaidi M, Vaughan L et al. Cytokines and immune cell phenotype in acute kidney injury associated with immune checkpoint inhibitors. Kidney Int Rep 2023;8:628–41. 10.1016/j.ekir.2022.11.020 36938084 PMC10014345

