# Supplementary material for: Synergistic effect of teclistamab with PD-1 inhibition: a case of acute interstitial nephritis with dual immunotherapy
Source: Clin Kidney J. 2024 Dec 20;18(2):sfae425. doi: 10.1093/ckj/sfae425 (PMC11836724; doi:10.1093/ckj/sfae425)
Supplement: sfae425_Supplemental_File [file sfae425_supplemental_file.docx]

**Supplementary Material**

**Supplementary Table S1**. Relevant laboratory investigations in the work up of acute kidney injury along with timeline of therapies

| Parameters | Reference  Range | D -22 | D -21 | D -18 | D -15 | D -13 | D -12 | D -11 | D -7 | D 0* | D 1 | D 2 | D 3 | D 4 | D 5 | D 6 | D 7 | D 14 |
| --- | --- | --- | --- | --- | --- | --- | --- | --- | --- | --- | --- | --- | --- | --- | --- | --- | --- | --- |
| **IV Dexamethasone 10 mg BID**  **Methylprednisolone 250mg daily**  **Teclistamab doses**  **Prednisone 60mg daily with taper dose**  **IV steroids and Tocilizumab for cytokine release syndrome** | | | | | | | | | | | | | | | | | | |
| Serum Creatinine (mg/dL) | **0.7 - 1.35** | **1.24** | **1.3** | **1.32** | **1.17** | **1.48** | **1.25** | **1.06** | **1.27** | **2.17** | **3.24** | **4.23** | **4.09** | **4.01** | **3.64** | **2.97** | **2.11** | **1.83** |
| Serum calcium  (mg/dL) | **8.8 – 10.2** | **9.1** | **3.0** | **8.9** | **8.7** | **8.2** | **8.0** | **7.9** | **9.0** | **8.4** | **7.8** | **7.7** | **7.5** | **7.5** | **7.4** | **8.0** | **8.1** | **8.1** |
| Serum C-reactive protein (mg/L) | **< 5.0** | **-** | **3.0** | **-** | **36.4** | **91.5** | **122.5** | **63.8** | **-** | **< 3.0** | **< 3.0** | **< 3.0** | **< 3.0** | **< 3.0** | **< 3.0** | **< 3.0** | **< 3.0** | **-** |
| Serum Kappa Free Light Chain (mg/dL) | **0.33 -1.94** | **36.9** | **-** | **-** | **53.4** | **65.3** | **-** |  | **49.0** | **65.3** | **-** | **62.2** | **51.8** | **-** | **-** | **-** | **-** | **-** |
| TNF – α  (pg/mL) | **< 10.0** |  |  |  |  |  |  |  |  |  | **60.1** |  |  |  |  |  |  |  |
| sIL-2R alpha  (pg/mL) | **< 959** |  |  |  |  |  |  |  |  |  | **>4000** |  |  |  |  |  |  |  |
| IL – 6  (pg/mL) | **< 5.0** |  |  |  |  |  |  |  |  |  | **75.6** |  |  |  |  |  |  |  |
| Urine retinol binding protein to creatinine ratio (mcg/g) | **<190** |  |  |  |  |  |  |  |  | **21212** |  |  |  |  |  |  |  |  |
| Urine protein to creatinine ratio (mg/g) |  |  |  |  |  |  |  |  |  | 0.89 |  |  |  |  |  |  |  |  |
| Investigations with negative results: Red blood cells in urine and titers for anti-nuclear antibody, anti – myeloperoxidase antibody, anti – proteinase 3 antibody, hepatitis B surface antigen, serum complement C3 and C4; Human immunodeficiency virus – 1 & 2 antibody screen. | | | | | | | | | | | | | | | | | | |
| *Day 0 corresponds to the day of diagnosis of acute kidney injury.  TNF – Tumor necrosis factor; IL – Interleukin; sIL-2R – soluble IL-2 receptor. | | | | | | | | | | | | | | | | | | |

**Supplementary Figure S1.** **Histopathology images from kidney biopsy**

**Figure S1.** a. PAS stain. Representative image of light microscopy shows acute tubular injury with focal interstitial inflammatory infiltrates in the absence of atypical myeloma casts. Insert shows severe tubulitis. b.  Immunohistochemical staining with CD3 highlights inflammatory infiltrates predominantly composed of T lymphocytes. c.  Immunofluorescence studies for kappa light chain illustrate few kappa light chain-restricted casts. d.  Immunofluorescence studies for lambda light chain in the same focus shows negative staining in the tubular casts.

**Supplementary References:**

S1. Isik B, Alexander MP, Manohar S, et al. Biomarkers, Clinical Features, and Rechallenge for Immune Checkpoint Inhibitor Renal Immune-Related Adverse Events. *Kidney Int Rep*. Apr 2021;6(4):1022-1031. doi:10.1016/j.ekir.2021.01.013

S2. Nishimoto N, Terao K, Mima T, Nakahara H, Takagi N, Kakehi T. Mechanisms and pathologic significances in increase in serum interleukin-6 (IL-6) and soluble IL-6 receptor after administration of an anti–IL-6 receptor antibody, tocilizumab, in patients with rheumatoid arthritis and Castleman disease. *Blood*. 2008;112(10):3959-3964. doi:10.1182/blood-2008-05-155846.

S3. Ratajczyk K, Konieczny A, Czekaj A, et al. The Clinical Significance of Urinary Retinol-Binding Protein 4: A Review. *Int J Environ Res Public Health*. Aug 11 2022;19(16)doi:10.3390/ijerph19169878.
